# Brain fog symptoms in individuals with and without post COVID-19 condition: translation and validation of the brain fog scale

**DOI:** 10.3389/fpsyt.2026.1681452

**Published:** 2026-03-06

**Authors:** Maria Loizidou, Ioulia Solomou, Flora Nikolaou, Fofi Constantinidou

**Affiliations:** 1 Center for Applied Neuroscience, University of Cyprus, Nicosia, Cyprus; 2 Department of Psychology, University of Cyprus, Nicosia, Cyprus

**Keywords:** brain fog, cognitive impairment, COVID – 19, neurocognitive ability, post COVID – 19 condition

## Abstract

**Introduction:**

Brain fog describes a heterogenous symptom encompassing cognitive symptoms, mental fatigue and reduced mental clarity, particularly prevalent among individuals with Post COVID – 19 Condition (PCC). This study aimed to translate and validate the Brain Fog Scale (BFS), originally developed in Polish, among a Greek-speaking population and explore whether individuals diagnosed with PCC report significantly more brain fog symptoms, compared to those without PCC.

**Methods:**

The BFS was translated in Greek, using a forward – backward translation process and was administered online. Principal Component Analysis and Confirmatory Factor Analysis were run to assess factor structure.

**Results:**

A total of 602 individuals (76.6% female) completed the BFS, of which 36 had a self-reported diagnosis of PCC. Internal consistency for the entire sample was excellent, α = 0.96. The BFS largely retained its original three factor structure with little variability: (1) impaired cognitive acuity (α = 0.95), (2) inattentiveness (α = 0.92), (3) mental exhaustion (α = 0.84). A Mann-Whitney U test revealed that individuals diagnosed with PCC reported significantly more brain fog symptoms compared to those without PCC, *U* = 2178.50, *p* = .011. MANOVA analyses further indicated significantly higher scores in the impaired cognition Factor among individuals with PCC, *F*(1, 125) = 7.32, *p* = .008.

**Conclusion:**

The BFS comprises a valid tool for assessment of brain fog and can facilitate person-centred rehabilitation planning in PCC. Findings are discussed in relation to the literature regarding brain fog symptom burden in PCC with suggestions for future research made.

## 1 Introduction

‘Brain fog’ is thought to be a core symptom of Post COVID – 19 Condition (PCC) (1), yet ‘fogginess’ or ‘clouding’ of the brain had been reported in the literature even before the COVID – 19 pandemic. Brain fog may be thought of as an umbrella symptom, as it refers to a fluctuating state of cognitive impairment, which may include confusion, lack of concentration, or memory difficulties, with large individual heterogeneity in subjective experience (2). Reports of ‘brain fog’ have been reported as a result of chemotherapy treatment in cancer patients (3), and in autoimmune conditions such as lupus (4), postural orthostatic tachycardia syndrome (5) and chronic fatigue syndrome (6), years before the COVID – 19 pandemic. Brain fog is thought to interfere significantly with daily functioning. For instance, in a cohort of 138 young adults with postural orthostatic tachycardia syndrome, brain fog was perceived to interfere with daily occupational and social functioning in most of the respondents, with 91% experiencing memory difficulties, and 88% reporting cloudy thinking, word finding difficulties and difficulty focusing (5).

Yet, it is the frequent occurrence of brain fog among individuals living with PCC (7, 8) that led to a global research effort to further our understanding of this heterogenous symptom. Presence of brain fog is positively correlated with worse clinical outcomes in COVID – 19 (9). Poorer memory and impaired multitasking ability, as part of brain fog symptomatology, were significant predictors of lower quality of life at work, among individuals with PCC (10). In a review of the possible pathogenic mechanisms of brain fog, authors discuss cellular metabolic processes, activation of microglia, a cascade of neuroinflammation, aggregation of tau proteins, or the development of autoimmune reactions as the result of virus-induced inflammation (11). Indeed, some evidence confirms the involvement of inflammatory markers, including interleukin (IL)-1B and IL-6, which are common in autoimmune conditions, at least in PCC – related brain fog (12). It has been argued that SARS-CoV-2 is involved in the alteration of several processes, including synaptic transmission and glycolysis therefore leading to cognitive symptoms such as memory loss or confusion (11). However, as the exact pathophysiology of brain fog continues to elucidate research, and in the absence of any reliable biomarkers, both researchers and clinicians rely on self-report measures for detection of the symptom.

Several self-report questionnaires have been developed aiming to reliably capture the multifaceted nature of brain fog. Ross and colleagues (5) attempted to characterise the symptoms and impact of brain fog among young people with postural orthostatic tachycardia syndrome, while also exploring management strategies. More recently, Knowles and colleagues (13) have developed and validated a brain fog scale for celiac disease, with a two factor structure capturing cognitive impairment, and somatic and affective experience. With relation to COVID – 19, the Brain Fog Scale (BFS) was developed to assess brain fog symptoms following COVID – 19 infection, with scale items also exploring cloudy thinking, word finding, and memory difficulties. (14). The BFS was originally developed in Polish and is currently being validated and standardised in the US population (15). The scale comprises of 23 self-report items and has three dimensions capturing “mental fatigue” (“I have felt fatigued”), “impaired cognitive acuity” (“I have found myself forgetting certain words such as the names of objects”) and “confusion” (e.g., “I have been daydreaming”) (14).

Therefore, although interest into the development and impact of brain fog has peaked after COVID – 19, presence of brain fog has affected the functionality and quality of life of various patient cohorts over the years. The large variability in individual experience and the absence of reliable biomarkers, turn our attention towards comprehensive self-report tools that can effectively capture patient experience. As such, translation and further validation of existing scales can ensure methodological consistency and improve the reliability of already developed tools. This research aims to: (a) translate and validate the English version of the BFS scale to the Greek – speaking adult population in Cyprus; (b) assess the psychometric properties of the Greek version of the BFS, including its reliability and validity, and (c) assess the levels of brain fog among individuals diagnosed with PCC. It is hypothesised that individuals with PCC will report significantly more symptoms on the BFS, compared to those who have not been diagnosed with PCC, rendering the BFS a useful clinical and research tool.

## 2 Materials and methods

This research was part of a larger scale study under the European Twinning initiative “Widening Participation & Strengthening the ERA”. The larger scale project “Brain Research and Integrative Neuroscience Network for COVID-19” is funded by the European Union’s Horizon Europe research and innovation programme under grant agreement no. 101079001 and has received ethical approval from the Cyprus National Bioethics Committee (EEBK/EΠ/2023/40).

### 2.1 Brain fog scale translation

Aligning with the operational definition by Debowska and colleagues (14), brain fog was defined as “a cognitive dysfunction characterised by problems with concentration and memory, inattention, confusion, difficulty understanding spoken and written language, reduced mental acuity, and mental fatigue”, (p. 2). Translation of the BFS in Greek was conducted following permission from the original author (14), using a blind forward- backward translation process.

Forward translation and cultural adaptation were completed by author ML, who is a native Greek speaker and proficient in the English language. A second member of the research team, also native Greek speaker (IS) completed the blind backward translation. Any discrepancies between the translations were resolved with consensus between the authors ML and IS. The Greek translation of the scale was further reviewed by senior author FC, native Greek speaker and proficient in English. Cultural adaptation of items included semantic clarifications which were necessary to preserve meaning, and conceptual unpacking to fit the Greek linguistic structures, such as separating “judgment” and “decision making” which in Greek may be understood as two distinct cognitive processes. It also included idiom translation for phrases such as “spaced out” which do not translate directly to the Greek language and required conceptual clarification.

The Greek version of the BFS comprised of 23 items and was scored on a 5-point Likert scale (0 = never, 1 = rarely, 2 = occasionally, 3 = a lot of the time, 4 = nearly all of the time). Participants were asked to indicate how often they experienced a given symptom in the past two weeks. Total scores range from 0 to 92, with higher scores indicating more severe symptoms.

### 2.2 Data collection procedure

The survey was developed on the Research Electronic Data Capture (REDCap) online platform (16). Participants were provided with information about the study objectives and procedure prior to deciding whether to consent. They were informed that their responses would be anonymous and that they could withdraw their participation at any point. Identifying information (e.g., name, city of residence, email) were not collected to ensure participant anonymity.

Mixed random sampling and snowball sampling procedures were used to achieve a sufficient sample size with diversity in COVID – 19 experiences. The study was advertised via social media platforms, including targeted posts in PCC community groups, and mailing lists. Participants over 18 years old were eligible to participate, but no other inclusion criteria were specified. Participants did not receive any credit for their participation. Data collection occurred over three phases. The first phase took place between October 2024 and November 2024, during which 146 participants completed the survey (Phase 1 dataset). As the sample size was insufficient, a second phase was conducted between December 2024 and February 2025 (Phase 2 dataset), yielding an additional 138 responses and a third data collection phase (Phase 3 dataset) took place between March 2025 and April 2025, yielding an additional 318 complete cases, to maximise sample size.

### 2.3 Survey development and measures

The survey was designed to be brief (under 10 minutes) and consisted of two main sections. The first section included basic sociodemographic information and COVID-19 infection history, while the second section comprised the BFS. In the second phase of data collection (Phase 2 dataset), additional clarifying questions were included regarding a having received a diagnosis of PCC from a physician, based on the WHO diagnostic criteria (World Health Organisation, 2021), and subjective COVID – 19 degree of recovery.

### 2.4 Participants

A total of 755 individuals accessed the online survey, with 602 providing informed consent to participate (Phase 1 dataset N = 146, Phase 2 dataset N = 138, Phase 3 = 318), resulting in a response rate of 79.74%. Out of 602 participants, 88.2% reported having contracted COVID – 19 at least once.

### 2.5 Data preparation

A total of 0.28% of values were missing overall. Despite their small percentage, Little’s MCAR test indicated that these values were likely not missing at random; X^2^ = 334.59, df = 232, *p* <.001). Careful exploration of missingness patterns revealed that this was driven by two cases who only responded to demographic questions and indicated that they had not contracted COVID-19 before. Listwise deletion of these cases reduced the percentage of missing values to 0.14%, and Little’s MCAR test was no longer significant, suggesting the remaining missing values were missing completely at random, X^2^ = 140.99, df = 154, *p* = .766.

### 2.6 Statistical analysis

Statistical analyses were completed on IBM SPSS Statistics (Version 29.0.1.0); Confirmatory Factor Analysis (CFA) was completed on IBM SPSS AMOS 29. Descriptive statistics, testing of assumptions and missing data analyses were conducted prior to any other analyses. Afterwards, a Principal Component Analysis (PCA) with direct oblimin rotation was used to extract the scale factors. To confirm the factor structure derived by PCA, a Confirmatory Factor Analysis (CFA) with Maximum Likelihood estimator was conducted. Adequacy of the sample size for factor analyses was assessed using the Kaiser-Meyer Olkin (KMO) measure. The Bartlett’s test of sphericity was used to assess intercorrelation of variables (17). Extraction of factors was based on the Kaiser criterion, thus factors with eigenvalues above one were retained (18). This was also confirmed via visual inspection of the scree plots. Reliability of the extracted factors was assessed using Cronbach’s alpha. CFA model fit was assessed using the X^2^ statistic, the Comparative Fit Index (CFI) (19) and the Tucker Lewis Index (TLI) (20), with values above 0.90 considered acceptable (21). The Root Mean Square Error of Approximation (RMSEA; (22) was also assessed to examine the error of approximation in the population (23).

To assess whether individuals with a PCC diagnosis experienced significantly more brain fog symptoms, an independent t-test was conducted on the Phase 2 dataset. PCC diagnosis was considered the independent, two-level variable, with BFS total scores used as the dependent variable. Group differences were also assessed for the extracted factors using a MANOVA analysis.

## 3 Results

### 3.1 Sample characteristics

Four-hundred and sixty-one participants (76.6%) were female. The largest age group was 18–29 years old, comprising 57.1% of the total sample, followed by the 40–49 age group at 23.8%. Out of 602 participants, 85.4% reported having contracted COVID – 19 at least once. Participant demographics are presented in **Tables 1**, **2**.

**Table 1:** Participant demographics for the entire sample.

Variable	Category	N (%)
Gender	Male	136 (22.6)
	Female	461 (76.6)
Age range	18–29 years old	344 (57.1)
	30–39 years old	84 (14)
	40–59 years old	143 (23.8)
	60+ years old	29 (4.8)
Education	≤ 12 years	223 (37)
	13–16 years	303 (50.3)
	17+ years	38 (6.3)
Occupation	Employed	303 (50.3)
	Unemployed	31 (5.1)
	Student	240 (40)
	Retired	16 (2.6)
	Other	7 (1.2)

**Table 2:** Participant COVID – 19 history.

Variable	Category	N (%)
Number of COVID – 19 infections	0	88 (14.6)
	1	213 (35.4)
	2	192 (31.9)
	3+	109 (18.1)
COVID – 19 recovery*	Full recovery	268 (68.9)
(self-report)	Substantial recovery	67 (17.2)
	Moderate recovery	35 (9)
	Little to no recovery	19 (4.9)
PCC diagnosis**	Yes	36 (27.7)
(self-report)	No	94 (72.3)

PCC, Post COVID-19 Condition. *N = 389; **N = 130.

The number of participants reporting a psychiatric condition increased from 21 (4.1%) prior to COVID-19 infection to 36 (7%) following infection. Descriptive statistics of health conditions, before and after COVID – 19 infections are presented in **Table 3**. Inspection of histograms indicated that data were normally distributed.

**Table 3:** Descriptive statistics of health variables before and after COVID-19 infection.

Health Condition	Prior to COVID-19 infection, N (%)*	After COVID-19 infection, N (%)*
Respiratory	50 (9.7)	41 (8)
Cardiovascular	33 (6.4)	30 (5.8)
Autoimmune/immune related	27 (5.3)	35 (6.8)
Neurological/cognitive	3 (0.6)	4 (0.8)
Renal (e.g., chronic kidney failure)	1 (0.2)	2 (0.4)
Liver/gastrointestinal	4 (0.8)	1 (0.2)
Endocrine/hormonal imbalance (e.g., thyroid)	50 (9.7)	38 (7.4)
Mental Health	21 (4.1)	36 (7)
Cancer	11 (2.1)	5 (1)
None of the above	314 (61.1)	322 (62.6)

*N = 514.

### 3.2 Principal factor analysis and confirmatory factor analysis

To extract BFS factors, a PCA with direct oblimin rotation was run on Phase 1 and 2 datasets only. Sample size adequacy (Kaiser-Meyer-Olkin = 0.947) and multivariate normality indices (Bartlett’s Test X^2^ = 5448.55, df = 253, *p* <.001) permitted the use of PCA. There was no singularity or multicollinearity of the correlation matrix, assessed using the determinant value. Based on the Kaiser criterion and following inspection of the scree plot, three factors were extracted; F1 explained 55.57% of variance (eigenvalue = 12.78), F2 explained an additional 7.56% of variance (eigenvalue = 1.74), and F3 explained further 5.86% of variance (eigenvalue = 1.35). Factor loadings are presented in **Table 4**. Correlations between factors ranged from 0.48 to 0.55. Internal consistency of the extracted factors was excellent to very good; F1 α = .95, F2 α = .92, F3 α = .84. Cronbach’s alpha for the entire sample was α = .96, indicating excellent internal consistency.

**Table 4:** Factor loadings from the three factors of the Brain Fog Scale (BFS).

Number*	Scale item (English and Greek)	PCA			CFA		
		F1	F2	F3	F1	F2	F3
1.	My thinking has been slow/ H σκέψη μoυ ϵίναι αργή	0.65			0.69		
4.	I have been easily distracted/ H πρoσoχή μoυ απoσπάται ϵύκoλα	0.52			0.69		
7.	I have found it difficult to take in and remember new information/ Bιώνω δυσκoλία στo να ϵισπράττω και να θυμάμαι νέϵς πληρoφoρίϵς	0.65			0.72		
8.	I have found myself forgetting certain words in a language that I know well, such as the names of objects/ Παρατηρώ ότι ξϵχνάω συγκϵκριμένϵς λέξϵις σϵ μια γλώσσα πoυ γνωρίζω καλά, όπως την oνoμασία αντικϵιμένων	0.77			0.63		
9.	I have found it difficult to think logically, which has affected my ability to make sound judgements and decisions/ Bιώνω δυσκoλία στo να σκϵφτώ λoγικά, τo oπoίo έχϵι ϵπηρϵάσϵι την κρίση μoυ και την ικανότητα μoυ να παίρνω απoφάσϵις	0.79			0.69		
10.	I have found it difficult to concentrate/ Δυσκoλϵύoμαι να συγκϵντρωθώ	0.62			0.77		
11.	I have found it difficult to think clearly/ Δυσκoλϵύoμαι να σκϵφτώ καθαρά	0.61			0.81		
12.	I have had a hard time finding the right words to express myself or explain things/ Δυσκoλϵύoμαι να βρω τις κατάλληλϵς λέξϵις για να ϵκφραστώ ή να ϵξηγήσω κάτι	0.84			0.79		
13.	I have found it difficult to formulate my thoughts/ Δυσκoλϵύoμαι να συντάξω τις σκέψϵις μoυ	0.79			0.78		
14.	I have felt like my mind has gone blank/ Nιώθω ότι τo μυαλό μoυ έχϵι αδϵιάσϵι	0.77			0.72		
15.	I have found it difficult to understand written words/ Δυσκoλϵύoμαι να κατανoήσω γραπτές λέξϵις	0.96			0.60		
16.	I have had difficulty understanding what other people say to me/ Δυσκoλϵύoμαι να κατανoήσω τι μoυ λένϵ oι άλλoι	0.82			0.63		
17.	I have been daydreaming/ Oνϵιρoπoλώ		0.85			0.53	
18.	I have felt spaced out, as if momentarily detached from reality/ Nιώθω αφηρημένoς/η, σαν να ϵίμαι στιγμιαία απoκoμμένoς/η από την πραγματικότητα		0.72			0.78	
19.	I have felt confused/ Nιώθω μπϵρδϵμένoς/η		0.57			0.86	
20.	I have experienced losing my train of thought/ Xάνω τoν ϵιρμό των σκέψϵων μoυ		0.42			0.78	
21.	I have felt lost/ Nιώθω χαμένoς/η		0.59			0.83	
22.	I have felt absent, as if I were living in my own world/ Nιώθω απών, σαν να ζω στoν κόσμo μoυ		0.76			0.80	
23.	My thoughts have been racing/ Oι σκέψϵις μoυ τρέχoυν		0.79			0.57	
2.	I have felt mentally exhausted/ Nιώθω πνϵυματική κόπωση			0.85			0.87
3.	I have felt drained/ Nιώθω ϵξαντλημένoς/η			0.87			0.91
5.	I have found myself getting annoyed/ Παρατηρώ ότι ϵκνϵυρίζoμαι ϵύκoλα			0.62			0.48
6.	I have felt sleepy during the day/ Nιώθω νυσταγμένoς/η κατά τη διάρκϵια της μέρας			0.60			0.68

*Number corresponds to the item number in the original scale. PCA, Principal Component Analysis; F1, impaired cognition; F2, inattentiveness; F3, mental exhaustion; CFA, Confirmatory Factor Analysis. All factor loadings are statistically significant at *p* <.05. The Brain Fog Scale is protected by copyright and should not be reproduced without written permission from the original author, Dr Agata Debowska. The authors of this paper confirm that they have obtained written permission to translate and validate the Brain Fog Scale in Greek.

The scale retained its original factor structure with little variability among individual scale items. However, to more accurate characterise the new factor structure, factor names were adjusted for clarity. Factor 1 labelled ‘impaired cognitive acuity’ contained 12 items, 9 of which were consistent with the original scale. Factor 2 labelled ‘inattentiveness’ contained seven items and was identical to the original scale (‘confusion’), except for item 16. Factor 3, ‘mental exhaustion’, contained four items, all of which were part of the ‘mental fatigue’ factor in the original scale.

CFA was run on Phase 3 dataset and confirmed the above factor structure (X^2^ = 1049,37, df = 227, *p* <.001); standardised factor loadings are presented in **Table 4**. The model demonstrated marginal fit to the data, RMSEA = 0.107, 90%CI [0.100, 0.113], CFI = 0.825, TLI = 0.787. However, these indices can be sensitive to smaller samples and should be interpreted with caution (24, 25). Although our sample size (*n* = 318) was adequate for CFA, some conservative approaches suggest a minimum requirement of 200 participants to minimise the possibility of estimation errors, which decrease as the sample size increases. A recent review of validation studies suggests that an overall sample of 300–500 participants may be adequate for studies utilising general population cohorts, despite the lack of clear guidelines on sample size for CFA (26).

### 3.3 Differences in brain fog symptomatology between-participants

To further explore whether individuals with PCC reported significantly more brain fog symptoms, compared to those without a PCC diagnosis, a non-parametric Mann-Whitney U test was run using only the Phase 2 dataset. This distinction was made due to the questions included in the Phase 2 dataset, regarding self-reported PCC diagnosis and degree of recovery, aligning better with the research hypotheses. The non-parametric equivalent was selected due to unequal group sizes between those diagnosed with PCC and those who did not have a PCC diagnosis. Therefore, in this analysis were included 130 cases. Scores on the BFS ranged from 2 to 87 (*M* = 38.47, *SD* = 19.57), with higher scores indicating higher symptom burden. There was a significant difference in responses on the BFS between participants diagnosed with PCC (81% females), who reported higher BFS scores, compared to those without a PCC diagnosis (82% females). This difference was statistically significant, *U* = 2178.50, *p* = .011.

To assess differences in participants’ responses in each of the BFS factors (impaired cognition; inattentiveness; mental exhaustion), a one-way MANOVA was run on the three dependent variables which were the standardised factor scores (impaired cognition, inattentiveness, mental fatigue), with PCC diagnosis as the independent variable. Testing of assumptions included testing for independence of residuals, multivariate normality, multicollinearity, and homogeneity of covariance matrices.

Q-Q plots and the Kolmogorov-Smirnoff test indicated that residuals were normally distributed. Box’s M and Levene’s tests were non-significant, suggesting homogeneity of variances and covariances. Using Wilk’s Lamda, there was a significant effect of PCC diagnosis on BFS factor scores, *V* = 0.93, *F*(3, 123) = 3.07, *p* = .030. Separate univariate ANOVAs explored the effect of PCC diagnosis for each of the extracted factor scores. There was a significant effect of PCC diagnosis on Factor 1 scores (impaired cognition), *F*(1, 125) = 7.32, *p* = .008. However, there was no effect of PCC diagnosis on Factor 2 (inattentiveness), *F*(1, 125) = 1.04, *p* = .310, or Factor 3 (mental exhaustion) scores, *F*(1, 125) = 0.54, *p* = .482.

## 4 Discussion

There has been increasing interest on the impact of brain fog on functionality and well-being both within clinical populations, but also among the general public (2, 27). This has boosted research into brain fog and the development of assessment tools, including self-report scales, to capture the heterogenous nature of this symptom. The present study translated and validated the Brain Fog Scale (BFS) originally developed by Debowska and colleagues (14), in the Greek language. Despite some variation, the Greek version of the BFS largely retained the original factor structure, with the presence of three factors: (1) impaired cognitive acuity, (2) inattentiveness, (3) mental exhaustion. These factors reflect the three dimensions theoretically and empirically proposed by Debowska and colleagues (14), referring to cognitive symptoms, mental fatigue, and concentration difficulties often experienced by individuals who report brain fog. The translated scale demonstrated excellent internal consistency, supporting its reliability and validity for clinical and research use among Greek-speaking populations. However, some variation in the item distribution was observed, specifically with items 1, 4, and 16, which were originally associated with the mental fatigue and confusion factors yet loaded onto the impaired cognition factor in the Greek version. This variation likely reflects cultural differences in how cognitive processes such as thought clarity, attention, and verbal comprehension are conceptualised in the Greek culture and are common concerns across the translation and validation literature (28, 29).

It was hypothesised that individuals diagnosed with PCC would score higher on the BFS scale, indicating higher symptom burden. This was based on an extensive body of literature reporting changes in cognitive acuity, concentration and clarity following COVID – 19 infection (7, 8, 11). Indeed, individuals diagnosed with PCC in the current sample reported significantly more brain fog symptoms relative to individuals without a PCC diagnosis. Further analyses revealed that this difference was only observed in responses given for the impaired cognition Factor. Therefore, individuals with PCC in the current sample reported significantly more symptoms relating to impaired cognition, than inattentiveness and mental exhaustion. A systematic review of the existing literature confirms a downward trend in symptom burden within the first 12 months of COVID – 19 recovery (30). Longitudinal evidence on the prevalence of memory loss, concentration loss and ‘cognitive blurring’ indicated that concentration loss was the least prevalent symptom by 18 months post COVID – 19 infection (31). Thus, it may be likely that cognitive symptoms are far more prevalent and pervasive, showing different rates of recovery in longitudinal assessments (31). This interpretation aligns with the moderate degree of symptom burden reported by our sample, contrary to the debilitating symptoms reported in the wider literature (7, 8). Although data collected for the purposes of this study did not include specific dates for infection and PCC diagnosis, the research team’s ongoing collaboration with physicians at the Cyprus Post-COVID Clinic suggests a very steep decline in the diagnosis of PCC over the past year across Cyprus, hypothesising that our participants are likely chronic patients.

Our findings bear critical clinical significance, particularly in the assessment and rehabilitation of individuals with PCC-related brain fog. The large individual variability in presentation suggested in the wider literature, was also evident by the large standard deviations obtained in this sample. Thus, comprehensive and personalised neuropsychological assessments are critical in ascertaining how a patient’s individual presentation may interfere with their capacity to engage with rehabilitation (32). This includes fatigue and cognitive complaints directly relevant to brain fog experience, or other PCC-related symptoms such as physical and neurological complaints, or mood disturbances (33). As such, neuropsychological assessment can include the BFS to help refine rehabilitation and treatment packages, responding to individual needs and thus improving the possibility for compliance with and efficacy of rehabilitation.

Lastly, it is important to consider that the sample comprised primarily of younger female individuals, most of whom were university students. This is common in online survey research, including COVID-19 related studies (34, 35). Gender distribution was similar across groups, with 81% females in the PCC group, and 82% females in the non-PCC group. It is well established that university students often present with higher levels of anxiety and depression, relative to the general population (36). This may have led participants without PCC diagnoses in the current sample, to score higher than they would have due to the presence of subclinical anxiety or depression symptoms. However, in the absence of any psychological assessments, it is difficult to assess this in the current sample.

### 4.1 Future directions

The development of self-report scales to capture the heterogenous symptoms of brain fog following COVID-19 infection is the first step in understanding the complex presentation of this condition. Future research should focus on biological and neurobehavioral biomarkers that can more accurately detect the presence of the symptom or assess individual risk for developing brain fog symptoms following viral infections. On this basis and considering the three theoretical constructs identified by Debowska and colleagues (14) in Polish, and also confirmed in the present study with a Greek cohort, research should explore potential brain fog phenotypes, which could differentiate between individuals with different clusters of symptoms. However, future research should also aim to further validate the Greek version of the BFS, using a larger and more representative sample based on *a priori* analysis.

## 5 Conclusion

Overall, this research aimed to translate and validate the BFS in a Greek-speaking adult population in Cyprus. The translated scale retained its original factor structure with the extracted factors being conceptually similar to those derived by Debowska and colleagues (14). Individuals diagnosed with PCC reported significantly more brain fog symptoms compared to those without PCC, as initially hypothesised.

## 6 Limitations

While there was representation of all age groups and variability in experiences with COVID – 19, the sample comprised largely of young, university-educated, healthy females. In the lack of other measures for anxiety or depression, in which younger, female individuals tend to score higher at, we acknowledge the potential bias of psychological states affecting our participants’ responses. Although our conclusions align with an extensive body of literature, future studies should aim for a larger and more representative sample. Onset of brain fog following COVID-19 infection was not specified as an inclusion criterion, and therefore it is possible that a portion of participants may have experienced brain fog as the result of conditions other than PCC. Whereas this may have diluted findings with regards to the brain fog burden of those with as opposed to without PCC, it is not expected to have affected the validation of the scale. Lastly, it should be noted that the cognitive complaints assessed by the BFS rely exclusively on self-report data and were not accompanied by objective measures of cognitive performance. Consequently, it is possible that these subjective complaints may not be fully captured by objective cognitive measures, and conversely, that objective cognitive impairments may not be reflected in self-reported symptomatology.

## Data Availability

The raw data supporting the conclusions of this article will be made available by the authors, without undue reservation.
